# Scalp Microbiota Dysbiosis in Seborrheic Alopecia and Restoration Following Herbal Extract Shampoo Intervention

**DOI:** 10.3390/microorganisms14051106

**Published:** 2026-05-13

**Authors:** Jing Feng, Jiancong Huang, Shaolu Zhou, Xianghai Chen, Gang Zhou, Xudong Wang, Xia Wen, Qingshan Shi, Pianjuan Guo, Qiongfei Li, Xiaobao Xie

**Affiliations:** 1 Guangdong Provincial Key Laboratory of Microbial Culture Collection and Application, State Key Laboratory of Applied Microbiology Southern China, Guangdong Detection Center of Microbiology, Institute of Microbiology, Guangdong Academy of Sciences, Guangzhou 510070, China; fjing@gdim.cn (J.F.); zgbees@gdim.cn (G.Z.); wangxudong@gdim.cn (X.W.); wenxia@gdim.cn (X.W.); jigan@gdim.cn (Q.S.); 2 Guangzhou Gotdya Fine Chemical Co., Ltd., Guangzhou 510805, China

**Keywords:** scalp, seborrheic alopecia, microbiota, herbal extract shampoo, intervention, LEfSe, *Staphylococcus*, *Malassezia*

## Abstract

Seborrheic alopecia (SA) is one of the most common forms of hair loss with a complex pathogenesis involving multiple etiological factors. Although the scalp microbiome has been implicated in various scalp disorders, its specific role in the development and progression of SA remains incompletely understood. To characterize the scalp microbiome in SA, we performed high-throughput sequencing of the 16S rRNA gene and ITS region on scalp samples from 41 Chinese SA participants before and after a 12-week intervention with a shampoo containing herbal extracts (ginger root, *Polygonum multiflorum*, and *Platycladus orientalis* leaf) and 29 healthy controls. The untreated SA group exhibited significant microbial dysbiosis compared to the healthy controls, characterized by reduced bacterial and fungal alpha diversity and increased relative abundances of *Staphylococcus*, *Cutibacterium*, and *Malassezia*. LEfSe analysis confirmed the significant enrichment of these three genera. Correlation network analysis revealed a substantial restructuring of microbial interactions in the untreated SA group: *Staphylococcus* and *Malassezia* lost all positive correlations with other genera, whereas *Cutibacterium* displayed relatively stable topological relationships. Following the 12-week intervention, the treated SA group showed significant clinical improvement (reduced hair loss and scalp sebum content), along with a restoration of microbial diversity to levels comparable to the healthy group and a normalization of the abundances of *Staphylococcus* and *Malassezia*. Our study confirms the critical role of scalp microecological dysbiosis in SA pathogenesis and identifies *Staphylococcus* and *Malassezia* as key taxa strongly associated with this dysbiosis. These findings provide a theoretical foundation for developing microbiome-targeted strategies for SA treatment and support the use of multi-targeted, plant-based interventions to restore microbial homeostasis and promote hair growth.

## 1. Introduction

Seborrheic alopecia (SA), also known as androgenetic alopecia (AGA) or male/female pattern alopecia (MPA/FPA), is a polygenic hereditary disorder characterized by androgen-dependent hair loss [1]. As the most common form of hair loss worldwide, SA can be diagnosed in both men and women, with a higher prevalence and more pronounced clinical presentation often exhibited in males, typically characterized by frontal hairline recession and vertex thinning [2]. Epidemiological data indicate that SA affects approximately 30% of middle-aged men around 30 years of age and more than 55% of older men over 50 years of age [3,4]. In females, the onset of SA is generally more gradual and occurs later in life. It is estimated that about 55% of women over 70 years of age are affected, with the prevalence increasing to up to 60% in White females by the age of 80 [4,5].

Clinically, SA is frequently associated with excessive scalp sebum production, oily hair, abundant dandruff formation, and varying degrees of pruritus [6]. Although SA is a chronic inflammatory skin disease resulting from the interaction of polygenic inheritance and environmental factors and is not life-threatening, its significant impact on physical appearance can lead to psychological distress [1,7]. Studies reported that 28.13% of SA patients experience symptoms of anxiety and depression [8]. Furthermore, a systematic review involving 2737 SA patients and 17,382 controls revealed that SA patients commonly exhibit increased levels of anxiety and depressive symptoms, along with reduced self-esteem, life satisfaction, emotional intelligence, optimism, and overall well-being [9]. These psychological burdens and associated impairments in social functioning have led to increased clinical attention toward SA.

The pathogenesis of SA is associated with multiple factors, including genetic predisposition, dysregulated 5α-reductase activity (leading to elevated dihydrotestosterone levels), heightened androgen receptor sensitivity, and disturbances in the local scalp microenvironment [1,3,6]. These factors collectively contribute to disruptions in the hair growth cycle (including a shortened anagen phase and prolonged telogen phase), changes in subcutaneous blood flow, sebaceous gland hyperplasia, and impaired function of hair follicle stem cells [3,5,6]. Notably, inflammation, abnormal sebum metabolism, and immune dysregulation are closely intertwined with these pathological processes, serving as key intermediaries in SA progression. Persistent low-grade inflammation impairs hair follicle stem cell viability, excessive sebum accumulation creates a pro-inflammatory microenvironment, and immune imbalance further exacerbates scalp tissue damage [4,10].

Current treatment strategies for SA include 5α-reductase inhibitors (e.g., finasteride), androgen receptor antagonists (e.g., spironolactone and bicalutamide), topical minoxidil, and complementary therapies such as traditional Chinese acupuncture and low-level laser irradiation, which can partially delay disease progression and promote hair growth [11,12,13,14,15]. However, treatment efficacy varies among individuals, and some medications may cause adverse effects such as sexual dysfunction [16]. Therefore, there is an urgent need to further investigate the underlying pathogenic mechanisms of SA, particularly regulatory factors beyond the classical androgen signaling pathway—such as the scalp microbiota, which has been reported to be closely linked to inflammation, sebum metabolism, and immune regulation, and may mediate SA progression [6,17,18].

Accumulating evidence indicates that the scalp microbiota plays a critical role in maintaining scalp health. A balanced microbial community comprising bacteria such as *Staphylococcus* and *Cutibacterium*, as well as fungi such as *Malassezia*, helps maintain the scalp’s barrier function and acidic pH environment [19]. Dysbiosis of the scalp microbiota has been associated with a range of scalp disorders, such as dandruff, seborrheic dermatitis (SD), scalp psoriasis (SP), and folliculitis [20,21,22]. In these pathological states, there is a significant increase in the abundance of specific pathogenic microorganisms, including *Malassezia* species (e.g., *Malassezia restricta*, *Malassezia slooffiae*, *Malassezia japonica*, *Malassezia furfur*) and bacteria such as *Staphylococcus epidermidis* and *Staphylococcus aureus* [23,24,25]. These microbes can exacerbate inflammation, disrupt epidermal barrier integrity, and lead to symptoms such as itching and scaling through mechanisms including the release of irritating metabolites [26,27,28,29].

Although the association between microbial dysbiosis and inflammatory scalp disorders like SD has been well documented, with *Malassezia* and *Staphylococcus* reported as key microbial players in SD [22], the precise role of microbiota in the initiation and progression of SA remains insufficiently studied. While SA shares some clinical features with SD such as sebum overproduction, it is a distinct entity primarily characterized by progressive hair follicle miniaturization rather than overt cutaneous inflammation [6]. Therefore, directly extrapolating findings from SD microbiome studies to SA may be inadequate, as the unique microbial signatures in SA and their functional implications in the context of hair follicle biology and the androgen-driven pathway warrant dedicated investigation. Furthermore, existing investigations into the scalp microbiome in SA remain limited and have predominantly employed cross-sectional designs [30,31,32]. These approaches cannot reflect dynamic changes in the microbiota during disease progression or in response to treatment. Considering the crucial functions of the scalp microbiota in maintaining skin barrier integrity, regulating local immune responses, and stabilizing the acidic microenvironment, we hypothesize that SA may not be solely a sebum-driven inflammatory condition but more likely a complex microbiota-host interaction disorder characterized by scalp microecological imbalance. Specifically, this imbalance may involve the abnormal proliferation of specific pathobionts and the disruption of microbial community structure and functional networks.

To test this hypothesis, a longitudinal cohort study was conducted utilizing 16S rRNA gene and ITS region amplicon sequencing to comprehensively analyze the scalp bacterial and fungal communities of 70 individuals from Guangzhou, China. The cohort comprised 41 SA participants, who were evaluated during both the active disease phase and after a 12-week intervention with an herbal extract shampoo, along with 29 healthy controls. The primary objectives of this study were: (1) to characterize the SA scalp microbiome and compare its diversity and composition with those of healthy individuals; (2) to identify key characteristic microbial taxa; (3) to evaluate changes in the scalp microenvironment and microbial community structure in SA participants before and after the intervention and to reveal microbiota remodeling in response to the intervention; and (4) to construct microbial co-occurrence networks to elucidate dynamic changes in community structure. This longitudinal interventional design allows us to move beyond mere association and explore the dynamic relationship between the scalp microbiome and SA treatment response. It is anticipated that this study will provide novel insights into the microecological pathogenesis of SA and establish a theoretical foundation for developing microbiome-based diagnostic methods and targeted intervention strategies.

## 2. Materials and Methods

### 2.1. Subject Recruitment

This study was designed as an exploratory scalp microbiome cohort study. The sample size was prospectively estimated using G*Power software (v3.1.9.2) based on the independent samples *t*-test. Referring to Cohen’s criteria and the general sample scale of exploratory studies in this field, the effect size was set at d = 0.8 with a significance level of α = 0.05 and statistical power (1-β) of 0.8. The calculation indicated that a minimum of 26 participants per group was required. To account for potential sample loss and technical failure during experiments, the target sample size was increased by approximately 10%, with a planned recruitment of 30 participants for each group. Ultimately, a total of 29 healthy volunteers (healthy group) and 41 individuals with SA (SA group) were enrolled, with both groups meeting the minimum sample size requirement. To further validate the statistical reliability of the cohort, a post hoc power analysis was performed (see Section 2.9), which demonstrated that the statistical power for core inter-group differential indicators exceeded 0.80. This confirms that the present sample size is sufficient to effectively identify differences in scalp microbial community characteristics between the two groups.

During recruitment, the gender and age distribution were balanced between the two groups to reduce potential confounding factors. The healthy group consisted of 8 males and 21 females, aged 24–42 years, while the SA group included 12 males and 29 females, aged 24–43 years (Supplementary Table S1). All participants had resided in Guangzhou, China for over one year. The study was conducted from April 2024 to April 2025. The study was approved by the ethics committee of the Institute of Microbiology, Guangdong Academy of Sciences (Approval No. 2024CT17; 1 April 2024). Written informed consent was obtained from all participants before their participation in the study. All experimental procedures were performed in accordance with the ethical standards of the institutional and national research committees and with the principles of the Declaration of Helsinki (revised in 2013). All data collected were kept confidential and used solely for the purposes of this research.

### 2.2. Inclusion and Exclusion Criteria

The diagnostic criteria for SA were based on the criteria outlined in “China Clinical Dermatology” [33]. The inclusion criteria for SA subjects followed the “Test method for efficacy measurement of anti-hair loss cosmetic products” [34] and were specified as follows: (1) age between 18 and 60 years, with no gender restriction; (2) hair length ranging from 5 to 40 cm; (3) gradually increasing hair shedding accompanied by mild hair thinning; (4) hair loss count exceeding 10 strands (all with visible hair follicles) using the 60-time combing method; (5) no chemical hair treatments, such as dyeing, perming, or styling, within one month prior to enrollment; and (6) willingness and ability to provide written informed consent.

The exclusion criteria were defined as follows: (1) pregnant or lactating women, or those planning pregnancy during the study period; (2) individuals diagnosed with severe androgenetic alopecia, alopecia areata, inflammatory scarring alopecia, or other scalp-related dermatological conditions; (3) individuals who had used antibiotics or medications that could influence study outcomes within the past two months; (4) individuals with psychiatric or psychological disorders, chronic sleep disturbances, or impaired emotional regulation; (5) individuals who had used anti-hair loss or hair growth-promoting cosmetics or related products within the past three months; (6) individuals who had taken systemic or topical agents known to affect hair growth within the past six months; (7) individuals with a history of hair transplantation; and (8) individuals with known allergic tendencies or heightened cutaneous sensitivity.

### 2.3. Scalp Intervention Protocol for SA Participants

Participants in the healthy group received no additional intervention. Participants in the SA group were provided with an herbal shampoo (Gotdya Fine Chemical, Guangzhou, China) containing extracts of ginger root (*Zingiber officinale*), *Polygonum multiflorum*, and *Platycladus orientalis* leaves for scalp intervention. They were instructed to use the shampoo according to their daily cleaning habits for 12 weeks. Usage duration and any discomfort or adverse responses were recorded throughout the experimental period. After 12 weeks, the 60-time combing test was performed on SA participants, and the number of shed hairs was recorded.

It should be noted that this shampoo is a commercially formulated product; thus, the proprietary herbal extract preparation and manufacturing processes are confidential and unavailable to the authors. In addition, the specific active compounds within this formulation were not analytically quantified in the current study. Nevertheless, the primary bioactive constituents of these herbal materials, including terpenes, flavonoids, stilbenes, and volatile compounds, have been well characterized in prior investigations. These components exert well-established anti-inflammatory, antioxidant, and anti-androgenic properties, which collectively provide a reasonable pharmacological basis for the current intervention.

### 2.4. Standardization of Hair Care and Daily Lifestyle Practices

To enhance the internal validity of the study and ensure that the observed effects could be attributed to the interventional shampoo, key potential confounding factors, which included hair care practices, dietary habits, and environmental exposures, were systematically controlled through participant instruction and protocol standardization.

During the screening and intervention phases of the trial, all participants were required to adhere to the following protocol: (1) refrain from washing their hair within 48 ± 4 h before each scheduled assessment, maintaining a consistent wash-free period before all evaluations; (2) avoid combing or brushing hair on the day of assessment; (3) maintain the same hairstyle as documented at baseline throughout all follow-up visits; (4) refrain from any hair care procedures (e.g., dyeing, perming) or any other anti-hair loss or hair growth treatments; and (5) maintain their usual lifestyle and avoid any significant changes to their routine that could affect hair physiology, including but not limited to drastic dietary alterations, prolonged exposure to harsh environmental pollutants, and changes in hair care habits. Specifically, participants were instructed to maintain their habitual dietary patterns, sleep/wake cycles, and general levels of environmental exposure (e.g., avoiding prolonged, uncharacteristic exposure to severe pollutants) as reported at baseline. Significant deviations from these routines were to be reported. These measures were implemented to minimize fluctuations in scalp microecology and hair growth physiology caused by external variables, thereby helping to attribute any observed intervention effect to the use of herbal shampoo.

### 2.5. Hair Loss Count and Scalp Physiological Parameter Assessment

At each visit, the 60-time combing test was performed by a trained technician to collect shed hairs and the total hair count was recorded. The combs used in the procedure had the following specifications: moderate tooth density with inter-tooth spacing of 0.9–1.1 mm, tooth length of 2.0–3.0 cm, and a total length of no less than 10 cm (excluding the handle). To ensure data consistency, combs made of the same material and meeting identical specifications were used throughout the study. Scalp sebum content was measured using an MB560 Meibometer (Courage+Khazaka, Cologne, Germany). Scalp hydration level was assessed using the moisture probe of the DermaLab^®^ USB system (Cortex Technology, Aalborg, Denmark). Hair density in the vertex region was evaluated using a 0–7 point scale (Supplementary Table S2) according to the “Test method for efficacy measurement of anti-hair loss cosmetic products” [34], with higher scores indicating greater hair density.

### 2.6. Sampling of the Scalp Microbiota

All scalp sampling procedures were conducted in a controlled environment room maintained at 21 ± 1 °C and 50 ± 10% relative humidity. To minimize fluctuations in scalp physiology and environmental interference, all participants were required to acclimate to these conditions for at least 30 min prior to sampling. A standardized sampling area was defined by parting the hair along the scalp midline with a sterile comb, establishing a clearly visible approximately 10 cm line from the frontal hairline to the vertex within the parietal (mid-scalp) region.

Scalp specimens were collected using individually packaged, sterile rayon-tipped swabs. Immediately prior to use, each swab was moistened with 0.85% sterile saline solution. The moistened swab was then rubbed back and forth along the demarcated line with firm and uniform pressure for 60 s to ensure sampling consistency and adequate microbial biomass. For quality control, additional saline-moistened swabs were exposed to the ambient sampling environment for 60 s without scalp contact, serving as negative controls to monitor potential exogenous microbial contamination. A total of 111 scalp samples were collected in this study, including 29 from the healthy group (n = 29), 41 from the SA group before intervention (untreated SA group, n = 41), and 41 from the same SA group after a 12-week intervention with the shampoo product (treated SA group, n = 41).

Immediately after sampling, the swab head was detached and transferred into a dry, sterile 15 mL centrifuge tube in a one-sample-per-tube manner to ensure sample independence, with no transport medium added. To minimize changes in microbial composition, all samples were processed within 10 min after collection and immediately snap-frozen at −80 °C. All frozen samples were subsequently transported on dry ice to Biomarker Technologies Corporation (Beijing, China) for high-throughput sequencing analysis.

### 2.7. DNA Extraction, PCR Amplification and Sequencing

Genomic DNA from swab samples was extracted with the Magnetic Viral Genomic DNA Kit (Tiangen Biotech, Beijing, China) according to the manufacturer’s protocol. The V3-V4 hypervariable regions of the 16S rRNA gene were targeted for bacterial DNA amplification using universal primers 338F (5′-ACTCCTACGGGAGGCAGCA-3′) and 806R (5′-GGACTACHVGGGTWTCTAAT-3′) [35]. For fungi, the first internal transcribed spacer (ITS1) region was amplified using the primers ITS1F (5′-CTTGGTCATTTAGAGGAAGTAA-3′) and ITS2 (5′-GCTGCGTTCTTCATCGATGC-3′) [36]. The amplification reaction comprised initial denaturation at 95 °C for 5 min, followed by 25 cycles of amplification (denaturation at 95 °C for 30 s, annealing at 50 °C for 30 s, and elongation at 72 °C for 40 s), and a final elongation step at 72 °C for 7 min. The presence and quality of PCR products were confirmed using 1.8% agarose gel (Biowest, Barcelona, Spain) electrophoresis, followed by recovery and purification using the Monarch^®^ DNA Gel Extraction Kit (New England Biolabs, Ipswich, MA, USA). The concentration and quality of the amplified libraries were assessed using a Qsep-400 fluorometer (Bioptic Inc., New Taipei City, Taiwan, China). Finally, the qualified bacterial and fungal amplicon libraries were sequenced on the Illumina NovaSeq6000 platform (Illumina, San Diego, CA, USA) with paired-end sequencing at Biomarker Technologies Corporation. All raw bacterial and fungal sequences were deposited in the National Center for Biotechnology Information (NCBI) Sequence Read Archive (SRA) under accession numbers PRJNA1421120 for bacteria and PRJNA1422460 for fungi.

### 2.8. Processing and Analysis of Sequencing Data

Microbiome bioinformatics analyses were performed using QIIME2 (v2020.6) and R package (v2.15.3) [37]. Raw demultiplexed reads were imported into QIIME2. Quality filtering and primer removal were conducted using the Trimmomatic (v0.33) and Cutadapt (v1.9.1) plugins, respectively [38,39]. The processed sequences were then denoised, merged, and screened for chimeras with the DADA2 plugin to generate amplicon sequence variants (ASVs) [40]. ASVs with fewer than three reads were filtered out to minimize sequencing artifacts. Bacterial sequences were taxonomically classified using the QIIME2 feature-classifier plugin with the SILVA Database (Release 138) through the classify-sklearn naïve Bayes classifier [41,42]. Fungal sequences were analyzed similarly using the UNITE database (v8.0) [43]. Prior to microbial diversity analysis, all samples were rarefied to the minimum sequencing depth (37,648 reads for bacteria and 40,242 reads for fungi) to correct for differences in sequencing depth among samples. Alpha diversity (Shannon index, Simpson index, and observed species) was assessed and visualized as violin plots using rarefied data [44]. Beta diversity, based on Bray–Curtis distances calculated from the rarefied ASV count [45], was visualized using principal coordinate analysis (PCoA) and non-metric multidimensional scaling (NMDS) [46]. Relative abundance was calculated as the proportion of sequences assigned to a given taxon relative to the total sequences per sample. Correlation networks of bacterial and fungal genera were constructed based on Spearman’s rank correlation analysis. The top 30 most abundant genera that exhibited significant correlations (Spearman’s correlation coefficient |r| > 0.5, *p* < 0.05 with Bonferroni correction) were included in the network construction. Network visualization was performed using the iGraph package (v1.2.5) in R (v2.15.3).

### 2.9. Statistical Analysis

Wilcoxon rank-sum tests were used to compare the alpha diversity of bacterial and fungal communities between groups. The statistical significance in bacterial and fungal composition between groups in the PCoA and NMDS plots was assessed by permutational multivariate analysis of variance (PERMANOVA) using the adonis function with 9999 permutations [47]. For differential abundance analysis, the Mann–Whitney U test with Benjamini–Hochberg False Discovery Rate (BH-FDR) correction for multiple comparisons was employed. The Linear discriminant analysis effect size (LEfSe) algorithm was applied to identify biomarkers that were significantly enriched in different groups using the non-parametric Kruskal–Wallis test coupled with linear discriminant analysis (LDA score > 4.0) [48]. Paired-sample *t*-tests were used to analyze differences in hair loss count and scalp physiological indicators in the SA group before and after the 12-week intervention. To assess the adequacy of the enrolled sample size (healthy group, n = 29; SA group, n = 41), a post hoc power analysis was conducted. Using the non-parametric Mann–Whitney U test with a two-tailed significance level of α = 0.05, the statistical power for detecting inter-group microbiome differences was calculated. Detailed effect sizes (Cohen’s d) and corresponding statistical power values for bacterial and fungal alpha diversity indices, as well as the abundances of core microbial genera, are summarized in Supplementary Table S3.

## 3. Results

### 3.1. Variations in Bacterial Community Diversity Among Healthy, Untreated SA, and Treated SA Scalps

**Figure 1:**
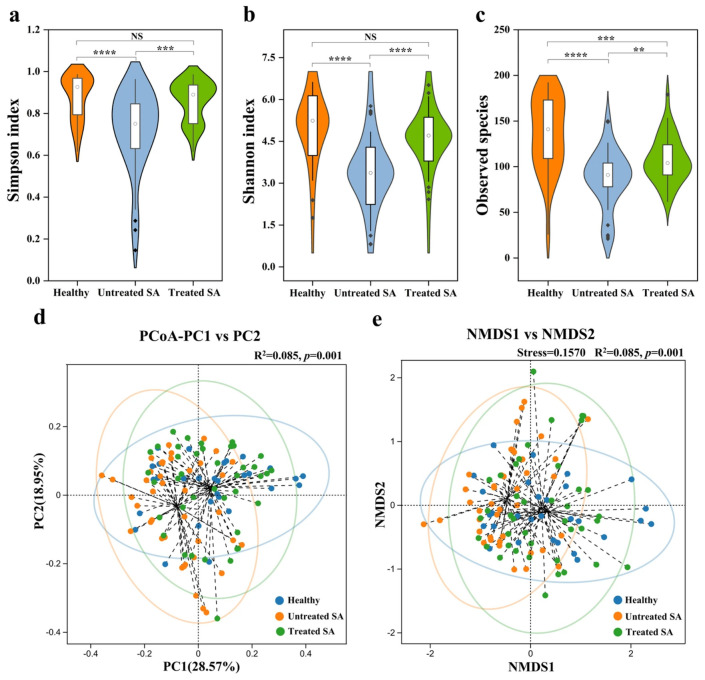
Comparative analysis of bacterial diversity between healthy scalps and SA scalps before and after intervention. Alpha diversity was assessed using the (**a**) Simpson index, (**b**) Shannon index, and (**c**) observed species index in healthy, untreated SA, and treated SA scalp samples. *p* values were calculated using Wilcoxon rank-sum tests. Beta diversity based on Bray–Curtis distances was evaluated using (**d**) PCoA and (**e**) NMDS analysis, with statistical differences among groups performed using PERMANOVA. Significant differences are indicated as follows: NS, not significant (*p* > 0.05); ** *p* < 0.01; *** *p* < 0.001; and **** *p* < 0.0001.

A total of 7,248,576 high-quality 16S rRNA gene reads were generated from 111 scalp samples using the Illumina NovaSeq platform, with an average of 65,302 reads per sample and a range from 37,648 to 75,689. In total, 1190 ASVs were obtained across all samples, with a median of 110 ASVs per sample (ranging from 27 to 194). Differences in bacterial community structure among healthy, untreated SA, and treated SA scalps were assessed using alpha and beta diversity analyses, as shown in Figure 1.

Alpha diversity analysis revealed significant differences between the healthy and untreated SA groups (Figure 1a–c). The untreated SA group showed significantly reduced diversity compared to the healthy group, indicating lower species richness and reduced community evenness. Specifically, the Simpson (0.72), Shannon (3.30), and observed species (87) indices in the untreated SA group were significantly lower than those in the healthy group (0.88, 4.90, and 136, respectively; *p* < 0.0001). After a 12-week intervention, the bacterial diversity of the treated SA group was significantly higher than that of the untreated SA group, with all three indices (0.86, 4.15, and 108) showing marked increases (*p* < 0.01). Moreover, no significant differences were found between the treated SA and healthy groups for the Simpson and Shannon indices (*p* > 0.05), whereas the observed species index remained significantly lower in the treated SA group compared to the healthy group (*p* < 0.01); importantly, the degree of this reduction was substantially smaller than that between the untreated SA and the healthy groups.

Beta diversity analysis based on Bray–Curtis distances revealed distinct clustering patterns among the groups. Both PCoA (R^2^ = 0.085, *p* = 0.001) and NMDS (stress = 0.1570, *p* = 0.001) ordinations showed that the bacterial communities of the healthy control and treated SA groups were closely positioned, while those of the untreated SA group formed a discrete, well-separated cluster. This clear separation indicated a robust difference in microbial community structure between the untreated SA group and the other two groups (Figure 1d,e).

### 3.2. Variations in Fungal Community Diversity Among Healthy, Untreated SA, and Treated SA Scalps

For ITS gene sequencing, 7,235,129 high-quality reads were obtained from 111 samples, with an average of 65,181 reads per sample (range: 40,242–73,173). A total of 1419 ASVs were identified across samples from the healthy and SA scalps before and after treatment, with an average of 60 ASVs per sample (range: 9 to 140).

**Figure 2:**
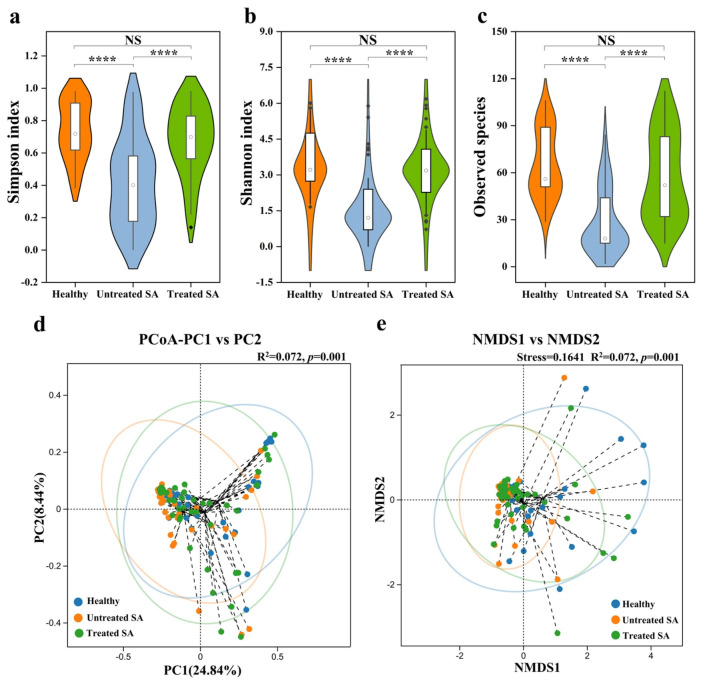
Comparative analysis of fungal diversity between healthy scalps and SA scalps before and after intervention. Alpha diversity was assessed using the (**a**) Simpson index, (**b**) Shannon index, and (**c**) observed species index in healthy, untreated SA, and treated SA scalp samples. *p* values were calculated using Wilcoxon rank-sum tests. Beta diversity based on Bray–Curtis distances was evaluated using (**d**) PCoA and (**e**) NMDS analysis, with statistical differences among groups performed using PERMANOVA. Significant differences are indicated as follows: NS, not significant (*p* > 0.05); **** *p* < 0.0001.

Alpha diversity analysis of the fungal community revealed that fungal diversity in the untreated SA group differed significantly from both the healthy and the treated SA groups (*p* < 0.0001; Figure 2a–c). The healthy group exhibited the highest fungal diversity, with the highest mean values for the Simpson (0.74), Shannon (3.68), and observed species (65) indices. In contrast, the untreated SA group showed the lowest values for all three indices (Simpson: 0.42; Shannon: 1.71; observed species: 28). Following the 12-week intervention, the treated SA group demonstrated a significant increase in fungal diversity compared to the untreated group, with higher values for the Simpson (0.67), Shannon (3.24), and observed species (58) indices. Although these indices remained slightly lower than those of the healthy group, no statistically significant differences were observed between the treated SA and healthy groups (*p* > 0.05), indicating a trend toward restoration of the fungal community structure after intervention.

Beta diversity analysis based on Bray–Curtis distances revealed significant differences among the three groups (PERMANOVA, *p* = 0.001; Figure 2d,e). Although some overlap was observed, PCoA showed that fungal communities in the healthy and treated SA groups clustered more closely together, while the untreated SA group formed a distinct cluster. This pattern was further supported by NMDS, which indicated clear separation of the untreated SA group from the other two groups.

### 3.3. Genus-Level Differences in the Scalp Microbiome Across the Healthy, Untreated SA, and Treated SA Groups

**Figure 3:**
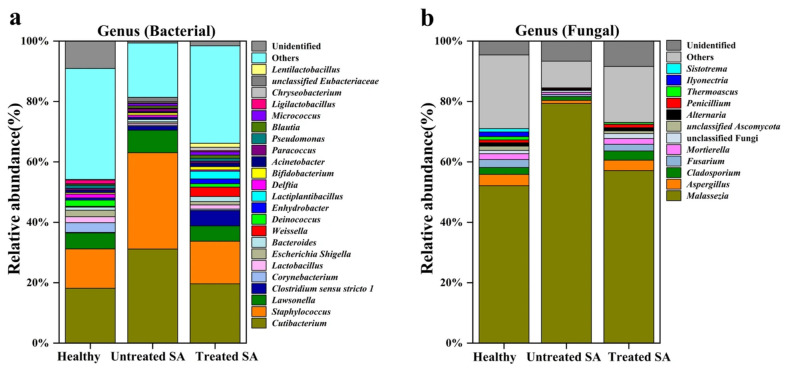
Taxonomic composition of bacterial (**a**) and fungal (**b**) communities at the genus level in the healthy, untreated SA, and treated SA groups.

To investigate the differences in bacterial and fungal microbiomes between healthy and SA scalps, the taxonomic composition of microbial communities at the genus level was analyzed among the three groups. Sequences assigned to the bacterial or fungal domain but that could not be classified into specific taxonomic groups were designated as “unclassified Bacteria” or “unclassified Fungi”. In contrast, sequences showing insufficient similarity to any reference sequences in the database were categorized as “unidentified”, indicating their absence from current taxonomic reference databases. The genus-level community structures for the three groups are presented in Figure 3, which displays only taxa with an average relative abundance greater than 1%, while the remaining taxa are grouped as “others”.

At the genus level, a total of 444 bacterial genera were identified. The predominant taxa (relative abundance > 5%) included *Cutibacterium*, *Staphylococcus*, *Lawsonella*, and *Clostridium sensu stricto 1* (Figure 3a). In the healthy group, the three most abundant genera were *Cutibacterium* (18.2%), *Staphylococcus* (13.0%), and *Lawsonella* (5.3%). Compared to the healthy group, marked increases in the relative abundances of *Cutibacterium* (31.1%), *Staphylococcus* (31.9%), and *Lawsonella* (7.4%) were observed in the untreated SA group, corresponding to increases of 71.4%, 144.8%, and 40.6%, respectively. In contrast, the relative abundances of *Corynebacterium*, *Lactobacillus*, *Escherichia-Shigella*, and *Deinococcus* were found to be reduced by 89.0%, 68.6%, 77.1%, and 92.4%, respectively. After 12 weeks of intervention, the relative abundances of *Cutibacterium* (19.6%), *Staphylococcus* (14.1%), and *Lawsonella* (5.1%) in the treated SA group were significantly lower than those in the untreated group and were close to the levels observed in the healthy group. Concurrently, the relative abundances of *Clostridium sensu stricto 1*, *Lactobacillus*, *Escherichia-Shigella*, *Bacteroides*, *Weissella*, and *Deinococcus* exhibited varying degrees of recovery toward healthy levels.

In the fungal community, 312 genera were detected, with *Malassezia* overwhelmingly dominant across all groups (Figure 3b). In the healthy group, *Malassezia* was found to have the highest relative abundance (52.1%), followed by *Aspergillus* (3.8%), *Fusarium* (2.6%), and *Cladosporium* (2.3%). Relative to the healthy group, an elevated *Malassezia* abundance (79.4%) was observed in the untreated SA group, reflecting a 52.3% increase, while the abundances of *Aspergillus*, *Cladosporium*, and *Fusarium* declined by 73.3%, 43.4%, and 74.9%, respectively. After 12 weeks of intervention, a reduction in *Malassezia* abundance to 57.1% was observed in the treated SA group, which was significantly lower than in the untreated group. Moreover, the relative abundances of *Aspergillus*, *Cladosporium*, and *Fusarium* increased, suggesting a partial restoration of the fungal community balance.

**Figure 4:**
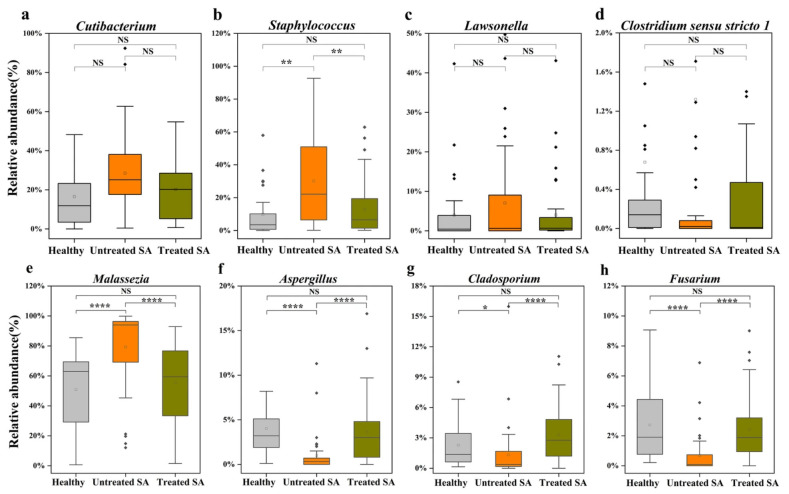
Differential abundance of high-abundance bacterial (**a**–**d**) and fungal genera (**e**–**h**) across healthy, untreated SA, and treated SA groups. (**a**) *Cutibacterium*, (**b**) *Staphylococcus*, (**c**) *Lawsonella*, (**d**) *Clostridium sensu stricto 1*, (**e**) *Malassezia*, (**f**) *Aspergillus*, (**g**) *Cladosporium*, and (**h**) *Fusarium*. Statistical significance of pairwise comparisons was determined using the Mann–Whitney U test with BH-FDR correction. Significant differences are indicated as follows: NS, not significant (*p* > 0.05); * *p* < 0.05; ** *p* < 0.01; and ****p < 0.0001.

To further analyze the differences in the dominant microbial communities among the three groups, statistical comparisons of high-abundance bacterial and fungal genera were conducted (Figure 4). Although the relative abundances of the bacterial genera *Cutibacterium*, *Lawsonella*, and *Clostridium sensu stricto 1* differed between the untreated SA group and the other two groups, these differences were not statistically significant (*p* > 0.05). In contrast, the abundance of *Staphylococcus* was significantly higher in the untreated SA group than in both the healthy and treated SA groups (*p* < 0.01), with no significant differences between the latter two groups (Figure 4a–d). Regarding the fungal community, the untreated SA group exhibited significantly enriched *Malassezia* and depleted *Aspergillus*, *Cladosporium*, and *Fusarium*. No significant differences in these fungal taxa were observed between the healthy and treated SA groups (*p* > 0.05) (Figure 4e–h).

Although significant fungal compositional alterations were detected for *Aspergillus*, *Cladosporium*, and *Fusarium*, their overall low relative abundances (<2%) suggest limited ecological contributions to scalp microecology. Accumulated evidence has confirmed that *Staphylococcus* and *Malassezia* act as core pathogenic microorganisms in multiple scalp disorders, including dandruff and SD. Accordingly, these two genera were defined as key signature taxa for subsequent analysis. The decline of minor fungal taxa is likely a secondary outcome driven by excessive *Malassezia* proliferation, representing an important feature of scalp microbial dysbiosis. Collectively, SA pathogenesis is closely associated with the overgrowth of pathobionts such as *Staphylococcus* and *Malassezia*, as well as the overall structural disruption of fungal communities. The competitive predominance of *Malassezia* and the concurrent loss of commensal fungi jointly contribute to scalp microecological imbalance in SA patients.

### 3.4. Distinct Scalp Microbiome Biomarkers Identified by LEfSe Analysis Across Healthy, Untreated SA, and Treated SA Groups

**Figure 5:**
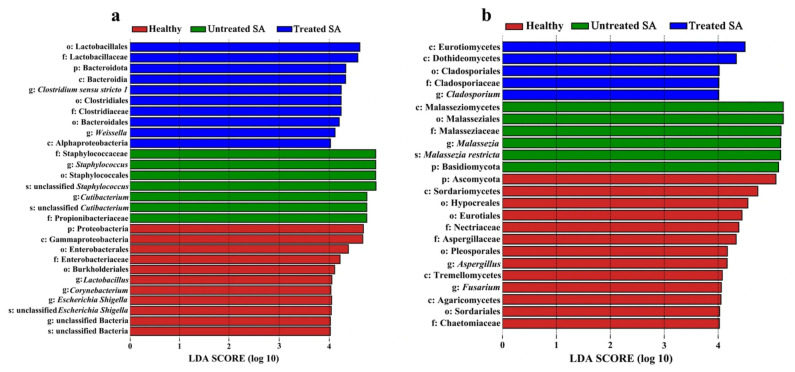
Bacterial (**a**) and fungal (**b**) taxa with significant differences among the healthy, untreated SA, and treated SA groups were identified by LEfSe analysis. Taxonomic abbreviations: p, phylum; c, class; o, order; f, family; g, genus; s, species.

To further investigate the differences in scalp microbiome community structure between the SA and healthy groups, linear discriminant analysis effect size (LEfSe) was performed with a threshold of LDA score > 4 (Figure 5). This criterion was applied to identify high-dimensional biomarkers and to assess the effect sizes of taxonomic units differentially enriched among the healthy, untreated SA, and treated SA groups.

A total of 28 differentially abundant bacterial biomarkers were detected from the phylum to the species level via LEfSe analysis, with 11 in the healthy group, 7 in the untreated SA group, and 10 in the treated SA group. For the fungal community, 24 biomarkers were identified from the phylum to the species level, including 13 in the healthy group, 6 in the untreated SA group, and 5 in the treated SA group. At the genus level, significant enrichment of two bacterial genera (*Staphylococcus* and *Cutibacterium*) and one fungal genus (*Malassezia*) was observed in the untreated SA group. In contrast, the healthy group showed enriched abundances of three bacterial genera (*Lactobacillus*, *Corynebacterium*, and *Escherichia-Shigella*) and two fungal genera (*Fusarium* and *Aspergillus*). Enrichment of two bacterial genera (*Clostridium sensu stricto 1* and *Weissella*) and one fungal genus (*Cladosporium*) was observed in the treated SA group. Furthermore, at the species level, significant enrichment of *M. restricta* was found in the untreated SA group, indicating a close association between this species and the pathogenesis of SA. However, due to the limited taxonomic resolution of the paired-end sequencing method employed, species-level bacterial identification was limited. Only unclassified *Staphylococcus* and unclassified *Cutibacterium* were detected as significantly enriched in the untreated SA group. Accordingly, the differential distribution of specific species within the genera *Staphylococcus* and *Cutibacterium* could not be further distinguished in the present work.

### 3.5. Correlation Network Analysis Reveals Key Roles of Staphylococcus and Malassezia in Scalp Microbiome Dysbiosis of SA Participants

**Figure 6:**
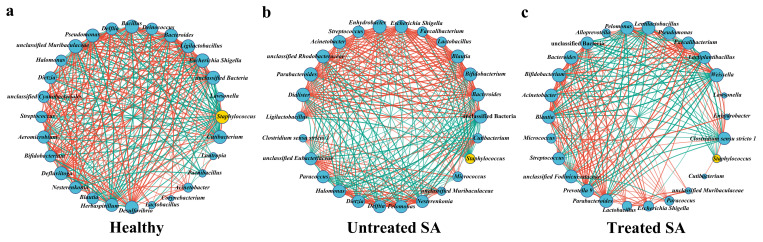
Bacterial co-occurrence network for the (**a**) healthy group, (**b**) untreated SA group, and (**c**) treated SA group. The genus *Staphylococcus* is highlighted in yellow. Line width is proportional to the strength of the Spearman correlation coefficient (|r| > 0.5, *p* < 0.05), with red lines indicating positive correlations and green lines indicating negative correlations.

**Figure 7:**
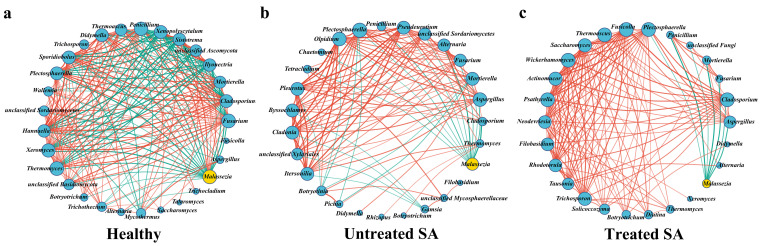
Fungal co-occurrence network for the (**a**) healthy group, (**b**) untreated SA group, and (**c**) treated SA group. The genus *Malassezia* is highlighted in yellow. Line width is proportional to the strength of the Spearman correlation coefficient (|r| > 0.5, *p* < 0.05), with red lines indicating positive correlations and green lines indicating negative correlations.

To elucidate the roles of highly abundant genera in the microbiome network of SA scalp, a co-occurrence network was constructed based on Spearman correlation analysis (|r| > 0.5, *p* < 0.05) using the top 30 genera in relative abundance. The results indicated that bacterial and fungal co-occurrence/co-exclusion relationships were significantly altered in the SA state, while partial restoration of network features was observed after the 12-week intervention (Figure 6 and Figure 7).

In the bacterial community, complex interaction patterns were exhibited by both *Staphylococcus* and *Cutibacterium* in the healthy group, characterized by multiple positive and negative correlations, reflecting a stable and balanced microecological structure. Specifically, *Staphylococcus* was found to have positive correlations with 7 genera, suggesting synergistic or commensal relationships, and negative correlations with 14 genera, indicating competitive or antagonistic interactions. *Cutibacterium* was observed to display positive correlations with 8 genera and negative correlations with 14 genera, indicating its broad involvement in diverse interspecific interactions within the healthy scalp microbiota (Figure 6a). In the untreated SA group, the microbial network was significantly disrupted. *Staphylococcus* underwent drastic changes, with all positive correlations disappearing and being replaced by negative correlations with all detectable genera (Figure 6b). This shift suggests a transformation in its ecological role from a complex participant with both cooperative and competitive interactions to a “disruptor” dominated by widespread competition. Such an extreme network structure implies that *Staphylococcus* may promote SA progression by suppressing the growth of other commensals and destabilizing the microbial community. In contrast, the interaction profile of *Cutibacterium* remained relatively conserved under SA conditions, retaining 8 positive correlations and 5 negative correlations. This conserved pattern contrasts sharply with the dramatic alterations observed in *Staphylococcus* and further suggests that *Cutibacterium* does not actively drive ecological imbalance or serve as a key pathogenic factor, but may function as a highly adaptable commensal.

After intervention, the bacterial interaction network in the treated SA group showed signs of recovery. *Staphylococcus* reestablished positive correlations with 2 genera, and its negative correlations decreased to 6 (Figure 6c), indicating a degree of reversibility in its network structure. These restorative changes further support the hypothesis that *Staphylococcus*-mediated ecological disruption is linked to SA disease activity. Meanwhile, *Cutibacterium* exhibited a noteworthy pattern, showing no significant correlations with any other bacterial genera. This differs substantially from its extensive interaction network in the healthy state (8 positive and 14 negative correlations) and its simplified but preserved interaction profile in the untreated SA group (8 positive and 5 negative correlations). This observation may reflect the dynamic characteristics and lagged recovery of the scalp microbial community during post-intervention restructuring, which suggests that the ecological reintegration of *Cutibacterium* may require an extended timeframe or depend on the prior reestablishment of other key microbial communities.

In the fungal community, *Malassezia* exhibited network dynamics similar to those of *Staphylococcus*. Under healthy conditions, it displayed positive correlations with 4 genera and negative correlations with 17 genera, forming a complex interaction network involving both cooperation and competition (Figure 7a). In the untreated SA state, *Malassezia* lost all positive correlations and showed negative correlations with all detectable fungal genera, although the number of negatively correlated genera decreased to 12. This change reflects a complete breakdown of mutualistic interactions and a shift toward a simplified, antagonism-dominated ecological dynamic (Figure 7b). Following the 12-week intervention, *Malassezia* still failed to re-establish any positive correlations; however, the number of negative correlations was reduced to 7 genera (Figure 7c). These results suggest that while the intervention effectively alleviated some ecological pressure and reduced the scope of antagonism, the reconstruction of cooperative networks within the fungal community lagged, indicating that ecological recovery in fungi may proceed more slowly than in bacteria.

### 3.6. A 12-Week Shampoo Intervention Reduces Hair Loss and Restores the Scalp Microenvironment of SA Participants

**Figure 8:**
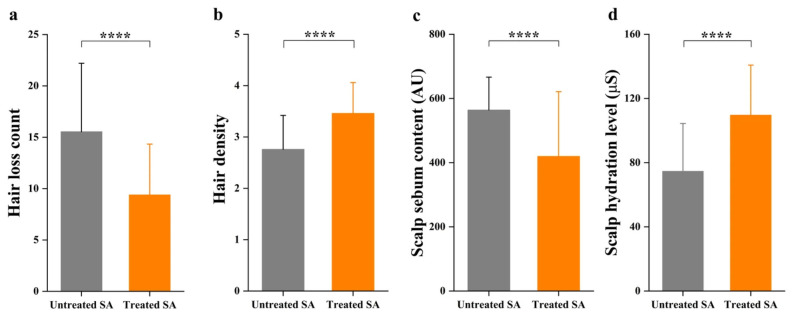
Changes in hair loss count and scalp physiological parameters following a 12-week shampoo intervention. Comparison of (**a**) hair loss count, (**b**) hair density, (**c**) scalp sebum content, and (**d**) scalp hydration level between the untreated SA and treated SA groups. Statistical significance was determined using paired *t*-tests (**** *p* < 0.0001).

To evaluate the effects of the herbal extract shampoo intervention on the scalp microenvironment, changes in hair loss count, hair density score, scalp sebum content, and scalp hydration level were monitored in SA participants before and after a 12-week intervention (Figure 8). Following the intervention, a significant reduction in hair loss (from 15.54 ± 6.67 to 9.39 ± 4.95, a decrease of 39.57%; Figure 8a) and a concurrent improvement in hair density score (from 2.76 ± 0.66 to 3.46 ± 0.60, an increase of 25.36%; Figure 8b) were observed. The scalp microenvironment was also markedly restored, as evidenced by a 25.59% reduction in sebum content (from 564.04 ± 102.48 AU to 419.71 ± 201.61 AU; Figure 8c) and a 46.93% increase in hydration level (from 74.62 ± 29.83 µS to 109.64 ± 31.19 µS; Figure 8d).

These physiological improvements indicate that the scalp microenvironment was effectively normalized. Excessive sebum secretion and impaired hydration are closely associated with the overgrowth of pathobionts and subsequent skin barrier dysfunction. Thus, SA symptoms were likely alleviated through the regulation of scalp physiological properties and the optimization of microbial habitats. Therefore, the therapeutic effect is likely mediated, at least in part, by the restoration of scalp microecological balance, as evidenced by the partial recovery of the microbial co-occurrence network structure, along with the marked reduction in the relative abundances of *Malassezia* and *Staphylococcus* in the treated scalp.

## 4. Discussion

SA is one of the most common forms of hair loss whose pathogenesis may involve scalp microecological imbalance. To investigate the role of the scalp microbiome in SA and evaluate the efficacy of the herbal shampoo containing extracts of ginger root, *P. multiflorum*, and *P. orientalis* leaves, dynamic changes in the scalp microbiome of SA participants before and after a 12-week intervention were analyzed and compared with those observed in healthy individuals. Significant dysbiosis in the scalp microbiome of untreated SA group was observed, characterized by reduced microbial community diversity and elevated relative abundances of *Malassezia* and *Staphylococcus*. Following 12 weeks of intervention, substantial microecological improvements were observed in the treated SA group, including reduced hair shedding, restored microbial diversity, and normalization of *Malassezia* and *Staphylococcus* abundances. These findings support the hypothesis that scalp microecological imbalance is involved in SA pathogenesis, with *Malassezia* and *Staphylococcus* being key components of this dysbiotic state.

The present findings confirm and extend prior cross-sectional reports from Asian cohorts, in which elevated abundances of *Malassezia* and/or *Staphylococcus* were consistently observed on the scalps of individuals with SA compared to healthy controls. For instance, an observation of the fungal microecology in hair follicles of Chinese SA patients found a higher *Malassezia*-positive rate in the alopecia group (60%) than in controls (40%) [49]. A microbiome study of Japanese male individuals with and without SA revealed dysbiosis in SA scalps, characterized by increased abundances of *Staphylococcus*, *Cutibacterium*, and *M. restricta*, and decreased abundances of *Corynebacterium* [31]. Similarly, an Indian cross-sectional study on female pattern hair loss (FPHL) showed significant differences in microbial composition between patients and healthy controls, with markedly reduced microbial diversity in FPHL participants correlating with the severity of hair loss [32]. The concurrent normalization of these key taxa and the reduction in hair shedding after treatment suggest that these microbes are likely active contributors to the disease process.

The concurrent overgrowth of *Malassezia* and *Staphylococcus* on SA scalps suggests that the two genera exert synergistic effects in SA pathogenesis. This synergy may drive scalp microenvironment imbalance and follicular damage by establishing a mutually reinforcing vicious cycle. These findings align with previously reported mechanisms: overgrowth of *Malassezia* can break down sebum, induce perifollicular micro-inflammation, and compromise the barrier function [49,50,51], whereas barrier dysfunction (often manifested as increased transepidermal water loss) is closely associated with enhanced colonization by *Staphylococcus* [52]. Notably, the intervention data obtained in this study further reveal the modifiability of this cycle: following treatment with the herbal shampoo, clinical hair loss symptoms improved concurrently with a synchronized reduction in the abundance of these two key pathobionts. This implies that the inflammation triggered by *Malassezia* and the barrier/pH homeostasis disruption mediated by *Staphylococcus* are not isolated events, but may rather constitute a “microecological pathogenic axis.” This axis is likely maintained through mechanisms such as metabolic cross-talk (e.g., staphylococcal hydrolysis products promoting *Malassezia* growth) and immune modulation (e.g., an inflammatory microenvironment weakening local immune surveillance) [29,53]. Therefore, combined targeting of this synergistic pathway, rather than individual microbial groups, may offer novel strategies for microbiome-targeted therapy for SA.

Microbial co-occurrence network analysis provided further insights beyond taxon abundance. The simplified and unstable interspecies network observed in the untreated SA group reflected disrupted microbial interactions and weakened community resistance to external disturbance. In contrast, partial restoration of network complexity post-intervention indicates that the herbal shampoo may aid in rebuilding a more robust and interconnected microbial community. This structural recovery, alongside the normalization of key taxa, implies that therapeutic efficacy may depend on restoring not only microbial composition but also healthy inter-species relationships, which are crucial for maintaining scalp homeostasis.

Furthermore, this study identified a unique ecological pattern of *Cutibacterium* in SA-related dysbiosis. Although the relative abundance of *Cutibacterium* was 31.1% higher in the untreated SA group, no statistically significant difference was detected compared with healthy controls. LEfSe analysis still identified *Cutibacterium* as a potential biomarker for the untreated SA group, which is consistent with previous reports of its enrichment in atrophic hair follicles of SA patients [18,31,32]. Given that SA scalps often exhibit excessive sebum secretion, the resulting lipid-rich microenvironment likely promotes *Cutibacterium* proliferation. Its increased abundance may therefore represent an adaptive response to the pathological condition rather than an indication of primary pathogenicity. This inference is strongly supported by microbial correlation network analysis, which showed that *Cutibacterium* maintained eight positive correlations with other taxa in both healthy and untreated SA groups, indicating a stable ecological niche largely resilient to disease status. In contrast, *Malassezia* and *Staphylococcus* showed a complete loss of positive correlations, decreasing from four and seven in healthy controls to zero in the untreated SA group, respectively. This suggests a profound restructuring of their ecological niches and underscores their central role in the microbial network disruption associated with hair loss. Thus, while the increased abundance of *Cutibacterium* may be linked to the pathological state, from a community-level functional perspective, it likely acts as a “follower” rather than a primary driver of structural network disruption.

Differential network characteristics from the treated SA group provide further evidence for this dynamic role. After 12 weeks of intervention, the overgrowth of *Staphylococcus* was effectively suppressed, and its intergeneric interactions gradually recovered from a competitive state toward positive correlations, consistent with a functional ecological reset of the microbial community [54]. Meanwhile, *Cutibacterium* exhibited a transient ecological island state in the treated SA group, with no significant positive or negative correlations with any other genera. This distinctive pattern clearly differs from both healthy and untreated groups and represents a key transitional signature of community recovery. Mechanistically, this phenomenon likely reflects the phased and delayed nature of community restructuring: strong perturbation dismantles prior interactions while a new stable network has not yet fully formed [55]. Such transient network simplification following disturbance is widely recognized as a transitional stage when microbial communities recover from dysbiosis toward homeostasis [56]. The temporary loss of *Cutibacterium* interactions indicates that the 12-week intervention not only modulated the abundance of key taxa but also deeply reshaped the dynamic rewiring of microbial relationships during ecological recovery. These findings reinforce the contribution of *Staphylococcus*-driven disruption to SA pathogenesis and provide an ecological perspective for understanding microbiome restoration after therapeutic intervention.

Given the critical role of microbial dysbiosis in the pathogenesis of SA, interventions targeting the restoration of scalp microecological balance represent a promising therapeutic direction. Herbal extracts, valued for their natural origin and multiple bioactivities, are being increasingly explored as adjunctive agents in cosmeceuticals for SA management [57,58]. In this study, a 12-week intervention with a shampoo containing extracts of ginger root, *P. multiflorum*, and *P. orientalis* leaves was found to significantly improve the scalp microecology in SA participants, as evidenced by reduced sebum secretion, increased hydration level, and partially restored microbial diversity. The abundances of key genera (*Malassezia*, *Staphylococcus*, and *Cutibacterium*), which are associated with inflammation, lipid metabolism dysregulation, and follicular imbalance, were substantially reduced to levels similar to those observed in healthy controls. These multidimensional results confirm the efficacy of this herbal extract shampoo in modulating scalp microecology and supporting hair growth.

Ginger root contains a variety of bioactive components such as terpenes, ketones, flavonoids, carotenoids, and alcohols. Recent studies have confirmed its potential in promoting hair growth [59]. The active constituent cedrol stimulates hair follicle cell proliferation and inhibits apoptosis by modulating the JAK3/STAT3 and Wnt3α/β-catenin signaling pathways, thereby ameliorating alopecia areata and promoting hair growth [60,61]. Meanwhile, ginger components also contribute to microecological modulation, zerumbone and 6-shogaol exert anti-inflammatory effects by suppressing TNF-α and IL-6, while ginger essential oil exhibits broad-spectrum antimicrobial activity against *S. aureus*, *S. epidermidis*, and some fungi [62,63,64]. *P. multiflorum*, a traditional Chinese medicinal herb used for hair growth promotion, is rich in various bioactive constituents such as stilbenes, anthraquinones, and flavonoids. Studies have demonstrated that *P. multiflorum* significantly increases hair follicle number and diameter in murine models, effectively ameliorating androgenetic alopecia [65,66,67]. The hair-promoting effects may be closely associated with its multiple bioactive constituents, such as (E)-2,3,5,4′-tetrahydroxystilbene-2-O-β-D-glucoside, torachrysone-8-O-β-D-glucoside, gallic acid ester of torachrysone-8-O-β-D-glucoside, and emodin-8-O-β-D-glucopyranoside [66,68]. Mechanistically, (E)-2,3,5,4′-tetrahydroxystilbene-2-O-β-D-glucoside and emodin-8-O-β-D-glucopyranoside suppress inflammatory responses via inhibiting the NF-κB signaling pathway [69,70]. Moreover, emodin and related anthraquinones show direct antifungal activity against *Malassezia*, a key pathogen in SA [71], which likely contributed to the reduced pathogenic microbial abundance observed in this study. *P. orientalis* leaves have long been used to reduce hair loss and promote hair regrowth [72]. Its extracts inhibit 5α-reductase activity and lower dihydrotestosterone levels, thereby counteracting androgenetic alopecia [73,74]. Its volatile oil and aqueous extract promote hair growth by stimulating dermal papilla cell proliferation, migration, and cell cycle progression [72,75]. Additionally, cedrol, a major active component of *P. orientalis*, suppresses IFN-γ-mediated inflammation and restores the immune microenvironment [76], while its essential oil has been reported to possess antimicrobial properties, collectively supporting scalp microecological balance.

It should be acknowledged that the lack of qualitative or quantitative analysis of specific active compounds in the shampoo formulation constitutes a limitation of this study, as it precludes the precise identification of the constituents directly responsible for the observed effects. However, as discussed above, the well-documented anti-inflammatory and antimicrobial properties of the key bioactive compounds in ginger root, *P. multiflorum*, and *P. orientalis* provide a coherent pharmacological basis for interpreting our clinical and microbiological outcomes, which include the reduction in pathogenic genera abundance, sebum secretion, and hair loss counts. Future research should focus on the identification, quantification, and investigation of synergistic mechanisms among these active compounds to further elucidate their specific molecular targets and pathways, thereby strengthening the mechanistic rationale and clinical translational potential of these findings.

Owing to the inherent limitations in taxonomic resolution of second-generation sequencing (SGS), only *M. restricta* was identified as enriched in the untreated SA group at the species level in this study. For bacteria, although differential enrichment of unclassified *Staphylococcus*, unclassified *Cutibacterium*, and unclassified *Escherichia-Shigella* was detected between the healthy and SA groups, precise species-level identification was not achieved. The paired-end sequencing method used in this study is a mainstream SGS approach. Although it improves accuracy through dual-end reading and sequence assembly, the typically short read lengths (< 500 bp) constrain taxonomic resolution [77,78]. Bacterial classification commonly relies on the 16S rRNA gene as a molecular marker. However, this gene is highly conserved among closely related species. For instance, the 16S rRNA gene sequences of multiple species within the *Staphylococcus* genus can exhibit over 99% similarity. Accurate species-level identification depends on discerning subtle genetic differences, which generally requires longer continuous sequences to cover sufficient variable sites. Under short-read conditions, it is challenging to effectively distinguish highly similar sequences, resulting in annotations often remaining at the genus level, with species frequently labeled as “unclassified.” In contrast, third-generation sequencing technologies (e.g., PacBio and Oxford Nanopore) overcome these limitations by generating read lengths of tens of thousands of base pairs, significantly improving genome assembly quality and enabling more precise discrimination of closely related bacterial species [79,80]. Therefore, to further elucidate species-level compositional differences in the scalp microbiome of SA, future studies should employ third-generation sequencing technologies integrated with multi-omics approaches, such as genomics and transcriptomics.

## 5. Conclusions

The scalp microbiomes of 41 SA participants were characterized before and after a 12-week intervention with an herbal shampoo and compared with those from 29 healthy controls. Significant microbial dysbiosis was observed in the untreated SA group, as evidenced by a marked reduction in microbial diversity and a significant increase in the relative abundances of *Malassezia* and *Staphylococcus*. Following the intervention, reduced hair loss and scalp sebum content were found in the treated SA group, alongside restored microbial diversity and normalized abundances of key scalp microbes to the levels observed in healthy controls. These findings underscore the association between scalp microbiome dysbiosis and SA, with *Malassezia* and *Staphylococcus* being highlighted as potential key microbial contributors and promising targets for SA management. Accordingly, strategies aimed at rebalancing scalp microbial homeostasis are recommended as an effective therapeutic modality for SA treatment. The favorable clinical and microecological outcomes achieved through this multi-target herbal intervention provide a solid experimental basis for its further development. Looking ahead, multi-omics strategies (microbiome, metabolomics, and transcriptomics) should be employed in future studies to elucidate the precise molecular mechanisms linking scalp microbial dysbiosis to SA pathogenesis. Furthermore, larger-scale and longer-term randomized controlled trials are warranted to validate the long-term efficacy and safety of microbiome-targeted herbal interventions across diverse populations. These efforts will deepen the understanding of host-microbe-disease interactions and pave the way for comprehensive SA management strategies based on scalp microecological regulation.

## Data Availability

The raw high-throughput sequencing data in this study have been submitted to NCBI Sequence Read Archive (SRA) (https://www.ncbi.nlm.nih.gov/sra) under accession number PRJNA1421120 for bacteria (Reviewer link: https://dataview.ncbi.nlm.nih.gov/object/PRJNA1421120?reviewer=onainlvomv8hbu4e3cq34odu1k, accessed on 10 February 2026) and PRJNA1422460 for fungi (Reviewer link: https://dataview.ncbi.nlm.nih.gov/object/PRJNA1422460?reviewer=8lj3hio3anmf5jetkumobls438, accessed on 11 February 2026).

## Supplementary Materials

The following supporting information can be downloaded at: https://www.mdpi.com/article/10.3390/microorganisms14051106/s1, Table S1: Distribution of gender and age among participants in the healthy group and SA group; Table S2: Hair density grading scale table; Table S3: Post hoc power calculation based on the Mann–Whitney U test for alpha diversity and core microbial genus relative abundance between healthy and untreated SA groups.

## Abbreviations

The following abbreviations are used in this manuscript:

SA	Seborrheic alopecia
AGA	Androgenetic alopecia
MPA	Male pattern alopecia
FPA	Female pattern alopecia
ASV	Amplicon sequence variants
PCoA	Principal coordinate analysis
NMDS	Non-metric multidimensional scaling
PERMANOVA	Permutational multivariate analysis of variance
BH-FDR	Benjamini–Hochberg False Discovery Rate
LEfSe	Linear discriminant analysis effect size
LDA	Linear discriminant analysis
FPHL	Female pattern hair loss
SGS	Second-generation sequencing
