# Supplementary material for: Scalp Microbiota Dysbiosis in Seborrheic Alopecia and Restoration Following Herbal Extract Shampoo Intervention
Source: Microorganisms. 2026 May 13;14(5):1106. doi: 10.3390/microorganisms14051106 (PMC13209585; doi:10.3390/microorganisms14051106)
Supplement: Supplementary file 1 [file microorganisms-14-01106-s001.zip › microorganisms-4251399-supplementary.pdf]

Supplementary material

Table S1. Distribution of gender and age among participants in the healthy group and SA group.

Groups	Gender	No. of participants	Ages	Mean ages
Healthy group	Male	8	24-34	27.50±5.68
	Female	21	24-42	28.29±3.98
SA Group	Male	12	24-36	29.50±4.32
	Female	29	24-43	29.90±4.09

Table S2. Hair density grading scale table.

Score	Description
0	No hair present
1	Extremely sparse, scalp clearly visible
2	Sparse, scalp easily visible
3	Moderately sparse, scalp visible
4	Medium density, minimal scalp exposure
5	Moderately dense, very little scalp visible
6	Dense, scalp faintly visible
7	Very dense, scalp almost invisible

Table S3. Post-hoc power calculation based on the Mann-Whitney U test for alpha diversity and core microbial genus relative abundance between healthy and SA-untreated groups.

Measured indexes	Specific indicators	Cohen's d (Effect size)	Statistical power (1-β)
Bacterial alpha diversity	Shannon index	0.954	0.92
	Simpson index	1.15	0.96
	Observed species	1.39	0.98
Fungal alpha diversity	Shannon index	1.31	0.97
	Simpson index	1.41	0.99
	Observed species	1.81	0.99
Core microbial genera relative abundance	*Cutibacterium*	-0.66	0.81
	Staphylococcus	-1.15	0.96
	Malassezia	-1.10	0.95

Note: Post-hoc statistical power analysis was performed based on the non-parametric Mann-Whitney U test with $\alpha = 0.05$ (two-tailed). Healthy group: n = 29, SA group: n = 41. Power ≥ 0.8 indicates sufficient sample size to reliably detect inter-group microbiome differences. Negative Cohen's d values represent the direction of difference, and absolute values were used for power calculation.
